# County-level association between modifiable health behaviors and premature mortality in the United States

**DOI:** 10.1016/j.pmedr.2025.103003

**Published:** 2025-02-10

**Authors:** Abdul Mannan Khan Minhas, Kashvi Gupta, Erin D. Michos, Andrew J. Sauer, Laurence Sperling, Vijay Nambi, Layla Abushamat, Christie M. Ballantyne, Dmitry Abramov

**Affiliations:** a Department of Medicine, Baylor College of Medicine, Houston, TX, United States; b Section of Cardiovascular Research, Baylor College of Medicine, Houston, TX, United States; c Department of Medicine, University of Missouri Kansas City, MO, United States; d Division of Cardiovascular Medicine, Johns Hopkins University School of Medicine, Baltimore, MD, United States; e Saint Luke's Mid-America Heart Institute, Kansas City, MO, United States; f Division of Cardiovascular Medicine, Emory Clinical Cardiovascular Research Institute, Emory University School of Medicine, Atlanta, GA, United States; g Division of Cardiovascular Medicine, Loma Linda University Medical Center, Loma Linda, CA, United States

**Keywords:** Premature mortality, Smoking, Sleep, Physical inactivity

## Abstract

Objective: Unhealthy lifestyle habits are associated with increased morbidity and mortality. This study aims to examine the county-level association of physical inactivity, insufficient sleep, and current smoking, three elements of the American Heart Association's Life's Essential 8, with premature mortality.

Methods: Premature country-level age-adjusted death rate (AADR) in 2018–2020 were obtained from National Center for Health Statistics Mortality Files. County-level data were included from County Health Rankings data set for 2022. Counties were divided into deciles based on the percentage of adults with physical inactivity, insufficient sleep, and current smoking (separate decile for each metric) from Behavioral Risk Factor Surveillance System in 2019. Multivariable linear regression were used to evaluate the association of premature AADR with physical inactivity, insufficient sleep, and current smoking (lowest deciles as reference), controlling for median county income as a social determinant of health.

Results: A total of 3082 counties were included in the analysis. Mean physical inactivity, current smoking, and insufficient sleep ranged from 20.7 % to 41 %, 13.4 % to 28.2 %, and 30.3 % to 43.7 % from 1st to 10th decile, respectively. There was a stepwise increase in premature mortality in each progressive decile with an increase in county-level physical inactivity, insufficient sleep, and current smoking, which remained significant in the adjusted analyses.

Conclusion: We demonstrate an independent association of county-level physical inactivity, insufficient sleep, and current smoking with premature mortality. Further research and public health efforts are needed to understand and mitigate these risk factors at the county level to improve health outcomes.

## 1 Introduction

Optimizing cardiovascular health is a national priority as cardiovascular disease is the leading cause of all-cause mortality in the United States (US). (Fang et al., 2016; Minhas et al., 2024)The American Heart Association lists eight essential health behaviors (Life's Essential 8, LE8) that constitute a heart-healthy lifestyle—including physical exercise, optimal sleep, and smoking avoidance—which are considered targets for intervention to optimize health at the individual level.(Lloyd-Jones et al., 2022) While causal effects of health behaviors on outcomes of individual patients have been well characterized and efforts to modify the behavior of individual patients are common, another approach to optimize population health may be through a focus on health behaviors at the regional level. Health behaviors vary at the county level as these are intricately linked with the built environment, food availability, and sociodemographic constitution of a county.(Hood et al., 2016) It remains unknown if variations in county-level modifiable health behaviors as identified by LE8 (such as physical activity, sleep, and smoking) are adversely associated with important public health outcomes. Therefore, we sought to evaluate whether county levels of physical activity, sleep, and smoking are associated with county mortality rates.

## 2 Methods

County-level data were included from the County Health Rankings data set for 2022. County-level physical inactivity, representing the percentage of adults aged 18 years and older reporting no leisure-time physical activity (age-adjusted), and county-level percentage of adults who are current smokers (age-adjusted) were obtained from the Behavioral Risk Factor Surveillance System (BRFSS) in 2019. County-level insufficient sleep represents the percentage of adults who report fewer than 7 h of sleep on average (age-adjusted), obtained from BRFSS in 2018. Premature mortality was reported as an age-adjusted death rate (AADR) among residents under age 75 per 100,000 population, obtained from the National Center for Health Statistics - Mortality Files 2018–2020. Premature mortality was defined as mortality under age 75 by this dataset to increase attention on mortality in younger patients which was potentially more preventable.(“Premature Death, 2025. County Health Rankings and Roadmaps”) Of the 3142 available counties in the dataset, 60 with missing/unreliable AADRs were excluded. Multivariable analyses further excluded one county missing data on median income. Counties were divided into deciles based on each health risk factor (separate decile for each metric). Multivariable linear regression, with beta (β) coefficients and 95 % confidence intervals (CI), were used to evaluate the association of premature AADR with physical inactivity, insufficient sleep, and current smoking (per decile [lowest deciles as reference] and as continuous variables). The β coefficients represent the mean difference (with CIs) in premature mortality with each decile of county prevalence of the health behavior. The multivariable model included physical inactivity, insufficient sleep, current smoking, and county median household income (as a social determinant of health). For example, the relationship between the deciles of smoking prevalence and AADR at the county level, as reflected by the β -coefficients, were adjusted for the prevalence of physical inactivity, insufficient sleep, and median county income.

All analyses were conducted using Stata 18.0 (Stata Corp.College Station, TX). The study met institutional guidelines and institutional review board approval for this study was not required, given the publicly available deidentified data.

## 3 Results

In our analysis, 3082 counties were included. The mean physical inactivity percentage from the 1st to 10th decile county rankings ranged from 20.7 % (standard deviation ±2.0) to 41 ± 2.8 %. Similarly, the mean insufficient sleep percentage from the 1st to 10th decile county rankings ranged from 30.3 ± 1.1 % to 43.7 ± 1.3 %. Finally, the mean current smoking percentage from the 1st to 10th decile county rankings ranged from 13.4 ± 1.8 % to 28.2 ± 2.3 %. Overall, the mean AADR ranged from 245.7 to 681.9 from 1st to 10th decile.

**Fig. 1:**
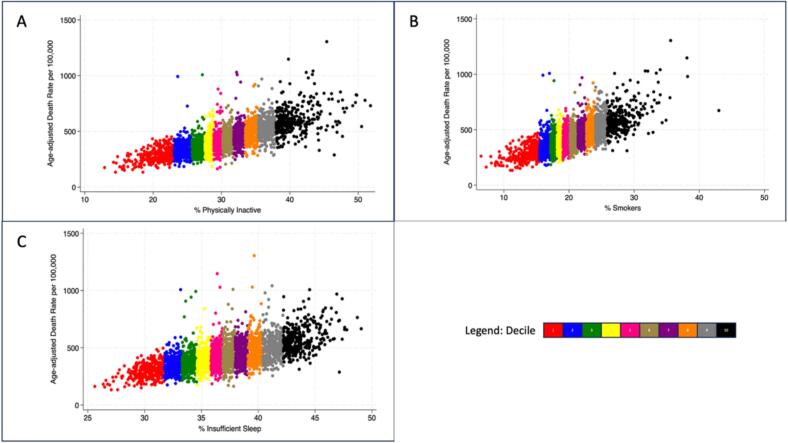
Relationship between county level prevalence of (A) physical inactivity, (B) current smoking, (C) insufficient sleep and premature age-adjusted death rate in 2018–2020 at the county level in the United States.

In multivariable models adjusting for each health behavior plus county median income, there was a stepwise increase in premature AADR with each progressive increase in deciles of county-level physical inactivity, insufficient sleep, and current smoking, as shown in Table 1. When used as continuous variables in univariable analyses, premature AADR increased with an increase in county-level physical inactivity (β-coefficient 16.1, 95 % CI 15.6–16.7), insufficient sleep (B-coefficient 20.3, CI 19.4–21.4), and current smoking (B-coefficient 21.8, CI, 21.1–22.6), Fig. 1. Each health risk factor remained independently associated with premature mortality in the adjusted model for physical activity, insufficient sleep, and current smoking: physical inactivity (β-coefficient 8.9, CI 8.2–9.6), insufficient sleep (β-coefficient 4.6, CI 3.6–5.6), and current smoking (B-coefficient 10.1, CI 9.1–11.1).

**Table 1:** The association between county prevalence of smoking, physical inactivity, and insufficient sleep (by deciles of county prevalence) and premature mortality in United States counties for 2018–2020.

Decile (Reference: 1)	Smoking β-coefficient⁎ (95 % confidence interval)	Physical Inactivity β-coefficient⁎ (95 % confidence interval)	Insufficient Sleep β-coefficient⁎ (95 % confidence interval)
2	29.6 (17.3–42)	9.2 (−3.3–21.7)	35 (23.1–46.9)
3	30 (16.8–43.1)	17.8 (4.7–31)	51.2 (39–63.4)
4	28.6 (15–42.2)	24.8 (11.2–38.3)	50.1 (37.6–62.6)
5	38.2 (24.4–52)	36.9 (23.2–50.6)	59.4 (46.5–72.2)
6	42 (27.7–56.4)	49.8 (35.6–64.1)	67 (54–80)
7	50.5 (35.5–65.6)	59.8 (45.1–74.5)	71.4 (58.1–84.6)
8	57.3 (41.7–72.8)	64 (48.5–79.4)	72.7 (59.1–86.4)
9	59.8 (43.4–76.2)	75.8 (59.7–91.9)	72 (57.8–86.2)
10	94.2 (76.8–111.7)	116.4 (99.3–133.5)	88.4 (73.2–103.6)

⁎ β-coefficient (95 % confidence interval) of age-adjusted death rate, using 1st decile of each health behavior as reference, with each health behavior β-coefficients adjusted for the other two health behaviors and median county income.

## 4 Discussion

In this analysis, we demonstrate that county-level lifestyle-related health behaviors such as physical inactivity, insufficient sleep, and current smoking are individually and independently associated with premature mortality. County-level variability in these health behaviors may explain some of the variability in all-cause mortality between counties in the US.

Previous studies have demonstrated the graded relationship between an individual's cardiovascular risk factors and mortality.(Lee et al., 2022; Yang et al., 2012) However, there are less data on the relationship between health behaviors and mortality at the county level.(Greer et al., 2016) Particularly, there has been a paucity of data on the independent relationship between the common health behaviors evaluated in the current analysis, each of which is a component of the American Heart Association LE8 health behaviors, and mortality at the county level. Therefore, our results on the independent relationship between health behaviors at the county level and premature mortality have important public health implications. While understanding an individual's behavior is important to address cardiovascular risk, targeting county-level interventions to address modifiable behaviors is also key because community-related factors significantly contribute to physical inactivity, insufficient sleep, and smoking risks. For example, physical inactivity has been associated with the built environment (sidewalks, playgrounds, parks, etc), climate, local amenities, and socio-demographic factors such as age, income, education, and race.(Creatore et al., 2016) Likewise, sleep quality and duration are impacted by socioeconomic factors with regional unemployment, lower education level, and other related factors.(Grandner et al., 2015) Finally, the rate of smoking at the county level can vary based on local, state, and federal policies including promotion of smoking cessation through community-wide education, treatment of nicotine dependence; therefore, policies that restrict access to nicotine-containing products may significantly improve health outcomes.(Sharbaugh et al., 2018) Consequently, this appreciation of county-level associations between these health behaviors and premature mortality may help to prioritize regional public health efforts that may underly unhealthy community behaviors, although optimal efforts to target both individual and community-level risk factors will require further evaluation. Furthermore, although multivariable adjustment for median county income as a social determinant of health attenuated the association between health behaviors and mortality, the relationship remained highly statistically and clinically significant, implying that addressing income inequalities in additional to other county factors may be important to optimize outcomes.

Our study has limitations. As this is a cross-sectional analysis, we cannot establish causality. Data for the variables included in the analysis were obtained from different years of the datasets based on availability. Other potential contributors to health behaviors at the county level, including those due socioeconomic differences beyond median income, are not evaluated in our analyses. These data are from a national US dataset and the relationship between health behaviors and outcomes in regions and countries outside the US will require further investigation.

## 5 Conclusion

In conclusion, county-level physical inactivity, insufficient sleep, and current smoking demonstrate an independent and graded association with county-level premature mortality. Populations in counties with higher modifiable behavioral risk factors and lower socioeconomic status have a more significant burden of premature mortality. An appreciation of the relationship between county-level health measures and outcomes can help guide public health efforts to identify at-risk counties and target interventions to improve the health of communities.

## Funding

none.

## CRediT authorship contribution statement

**Abdul Mannan Khan Minhas:** Writing – review & editing, Writing – original draft, Visualization, Formal analysis, Conceptualization. **Kashvi Gupta:** Writing – review & editing, Writing – original draft. **Erin D. Michos:** Writing – review & editing. **Andrew J. Sauer:** Writing – review & editing. **Laurence Sperling:** Writing – review & editing. **Vijay Nambi:** Writing – review & editing. **Layla Abushamat:** Writing – review & editing. **Christie M. Ballantyne:** Writing – review & editing. **Dmitry Abramov:** Writing – review & editing, Writing – original draft, Supervision.

## Declaration of competing interest

The authors declare that they have no known competing financial interests or personal relationships that could have appeared to influence the work reported in this paper.

## Data Availability

data are publicly available
